# Research on the design of panoramic virtual learning environment screen elements

**DOI:** 10.3389/fpsyg.2023.1314076

**Published:** 2024-01-08

**Authors:** Guan Huang, Li Zhou, Dan Chen, Wen Chen, Rui Liu

**Affiliations:** ^1^School of Education, China West Normal University, Nanchong, Sichuan, China; ^2^Department of Information Engineering, Southwest Jiaotong University Hope College, Chengdu, Sichuan, China; ^3^School of Cyber Science and Engineering, Sichuan University, Chengdu, Sichuan, China

**Keywords:** panoramic virtual learning environment, screen elements, eye-tracking technology, learning effect, interpretation method, caption

## Abstract

Panoramic video and virtual reality technologies create learning environments that provide learners with an “immersive” experience. In recent years, panoramic video design to create immersive learning environments, in particular, has become an increasingly popular topic in teacher education and educational research. However, few studies have explored the elements of panoramic virtual learning environment screens regarding the design of learning environments. Therefore, this experimental study uses eye-tracking technology to investigate how learners are guided by panoramic video elements in a panoramic virtual learning environment. Participants (*n* = 90) were randomly assigned to one of six conditions: (1) no caption + live interpretation, (2) no caption + AI interpretation, (3) 120-degree caption + live interpretation, (4) 120-degree caption + AI interpretation, (5) static follow caption + live interpretation, and (6) static follow caption + AI interpretation. The results of the study show that when learners experience a panoramic virtual learning environment with different narration methods, the live interpretation method is more likely to attract learners’ attention and bring better emotion and experience than the AI interpretation method. When experiencing a panoramic virtual learning environment with different caption presentation methods, the caption presentation methods induced learners’ attention, learning emotions, and experiences in the order of no caption >120-degree caption > static following caption. Finally, the rules for optimizing the design of panoramic virtual learning environment screens are given based on the findings of the study, which provide new ideas for designing and developing panoramic video teaching resources.

## 1 Introduction

Virtual reality (VR) learning experiences are engaging and allow learners to immerse themselves in content beyond what is possible in the real world (Rupp et al., 2019). Panoramic video based on VR technology is gaining increasing attention from educational researchers (Wen-hao et al., 2020). Panoramic video provides real 360-degree omnidirectional sequences, with pixels usually arranged in a spherical shape to provide an immersive experience for users. Panoramic video is the product of a combination of multiple video technologies. Such videos allow the audience to have a wider field of view and more realistic field of view experiences (Shang et al., 2022). While many studies have shown that panoramic videos are beneficial for learning, some evidence that panoramic videos are detrimental to learning has also emerged. For example, due to the 360° perspective of VR video scenes and the linear variation of the audio narrative, learners may be distracted or have difficulty focusing on the VR video learning process, resulting in the loss of important learning content and reduced learning effects (Ardisara and Fung, 2018; Shadiev et al., 2022). Therefore, this study used multimedia theory to explore the impact of panoramic video elements on learners and make suggestions for the design and development of panoramic video to provide learners with a better learning environment.

### 1.1 Application of panoramic virtual learning environments in education teaching

#### 1.1.1 Panoramic education videos in virtual learning environments

Researchers in many places have begun to explore the application of VR in teaching and learning (Radianti et al., 2020), but this research in the field of education currently focuses on the following four areas: VR to support the creation of learning environments, support skills training, support language learning, and support children’s education (De-Jian et al., 2016). An immersive virtual learning environment can help learners establish connections between new and existing knowledge and encourage learners to actively choose, organize, and integrate information. In addition, it enables meaningful learning (Araiza-Alba et al., 2021). Educational panoramic video is a new type of learning environment developed for educational teaching scenarios, where learning content is nested in the panoramic learning space, and learners can learn through body interactions with the environment, which affects immersion and fun. The presentation characteristics of panoramic video are conducive to the construction of highly efficient, personalized and open learning scenarios and hybrid learning spaces (Nan-Zhong, 2017).

#### 1.1.2 The significance of panoramic virtual learning environments in teaching and learning research

First, panoramic videos can be used as teaching resources in education. For example, T. Y. Yang and C. H. Huang claimed that 360-degree panoramic video is a positive way for nursing students to study physical examination. It provides a simple and practical way to practice motor techniques under realistic conditions. When teaching physical education, 360-degree video has the best effect (Yang et al., 2022). Mostafa Seifan argued that panoramic video can be used as a teaching resource in architecture classes to give learners authentic teaching and learning outcomes, allowing learners to make multiple connections to the real world through panoramic video in the classroom (Seifan et al., 2020). J. C. Domínguez suggested that simulation software is an excellent tool widely used in academic teaching to link theory and traditional practice. This work focuses on the use of panoramic video resources as a complement to traditional laboratories to study hydrogen production from electrolytic water in a virtual environment (Domínguez et al., 2018).

Second, education through panoramic video can be effective in improving the learning experience. An experimental study approach was used to design, implement, and evaluate a complete cycle of panoramic video embedded in two undergraduate classes, “Pharmacology and Therapeutics” and “Understanding Ecotourism,” and learners expressed higher learning satisfaction, as well as more opportunities to practice professional skills and explore historical artifacts with deeper cultural understanding (Hodgson et al., 2019). Jensen and Konradsen found that learners who use immersive headsets are more engaged; spend more time on learning tasks; gain better cognitive, psychomotor, and emotional skills; and have good learning experiences (Jensen and Konradsen, 2018). Some experts have also used special effects such as transitions and subtitles in postediting and editing to guide the attention of learners to choose the right spot in panoramic videos so that they can have the most comfortable learning experience and thus improve their learning performance (Yuan, 2020).

Third, panoramic video in education and teaching has shortcomings. For example, Schwarze A found that panoramic video viewing equipment may cause motion sickness in learners, which prevents learners from focusing on the learning content (Schwarze et al., 2019). The overly realistic and complex scenes in panoramic videos may distract learners’ attention to irrelevant content, leading them to be lost in the scenes, decreasing their interest in learning, decreasing their positive learning experience, and increasing their cognitive load, which ultimately reduces learners’ learning efficiency and affects their learning results (Rong-Huai and Yang-JunY, 2012). Therefore, designing the elements of panoramic video, giving full play to the advantages, and avoiding the shortcomings of panoramic video have become urgent problems in current research.

### 1.2 Panoramic virtual learning environment screen elements

Panoramic video is an emerging multimedia picture based on VR technology, and when designing its components, the research framework of multimedia learning needs to be followed; at the same time, panoramic video has special “fusion of reality and reality” characteristics, and effective design delivers visual highlights to learners and produces “new quality” in perception. In the cognitive theory of multimedia learning, multimedia involves the combination of “words” (words) and “pictures” (pictures) to present materials (Mayer, 2021). Collating existing studies shows that the constituent elements are the basic units of picture composition (Zhi-Jun and Xue, 2015). At present, the picture elements of panoramic video mainly include sounds, texts, annotations, pictures, images, and videos (Alpizar et al., 2020; Albus et al., 2021).

## 2 The objectives and working hypothesis of the current study

Based on the composition of panoramic virtual learning environment screen elements, this study investigates how to use the perceptual characteristics of learners to reasonably standardize panoramic video screens to enable learners to actively participate in multisensory, direct learning attention to key learning content and generate deep learning cognitive activities. Using a literature research method, survey research method, and experimental research method to investigate learners’ learning attention guidance, panoramic video was developed and applied in this study with 90 college learners in groups through eye-tracking technology to explore the changes in learners’ eye-movement indicators under different subtitle and narration presentation methods, which in turn illustrated how learners’ attention was guided. In addition, emotion and experience scales were used to measure learners’ emotions and experiences and to provide additional information on the change in learners’ emotions, thus increasing the data support.

A 3 (no caption, 120-degree caption, static follow caption) × 2 (live interpretation, AI interpretation) experimental design was used in this experiment. The same panoramic video resource was used for the six groups of experiments, and only the caption and interpretation design varied across groups, while the remaining screen content was the same, as shown in Table 1.

**Table 1:** Specific experimental items and experimental contents.

Research items	Factor level	
Effect of caption presentation mode and different interpretations on learners’ attention	1. No caption	Live interpretation
	2. No caption	AI interpretation
	3. 120-degree caption	Live interpretation
	4. 120-degree caption	AI interpretation
	5. Static follow caption	Live interpretation
	6. Static follow caption	AI interpretation

The independent variables of the experiment are the three levels of caption design in the panoramic video, i.e., no caption, 120-degree caption, and static follow caption; the narrative was presented as live interpretation or AI interpretation. The dependent variables were the eye-movement index (total fixation duration, number of whole fixations, first fixation duration, and number of saccades) and emotional indicators (learning emotion, experience). For the relationships among interpretation mode, subtitle presentation mode, learning attention, learning emotion, and experience, we propose the following hypotheses:

*Q1:* Does the interpretation method in panoramic videos affect learners’ attention, learning emotions, and senses of experience?

*H1:* When learners view panoramic videos, live interpretation is more likely than AI narration to attract their attention (H1a), increase their emotions (H1b), and yield a good experience (H1c). The underlying rationale for this hypothesis comes from the fact that AI narration suffers from a lack of emotion, which, in turn, affects the learning experience (Alejandro et al., 2021; Yingmin, 2023).

*Q2:* Does caption presentation mode in panoramic videos affect learners’ attention, moods, and experiences?

*H2:* When learners view panoramic videos, caption presentations are more likely to attract their attention (H2a), enhance their emotions (H2b), and yield good experiences (H2c) than presentations without captions. The rationale behind this hypothesis is that text subtitles help direct attention to relevant information and enable a timely repetition of narrative content (the Signaling Principle: Mayer, 2021). In addition, learners in cued conditions may attend to signaling elements more frequently (Wang et al., 2020).

## 3 Method

### 3.1 Participants

In this study, subjects were randomly recruited from X University through posters and other forms, with no restrictions on age or major. A total of 126 subjects were recruited. Because this experiment required wearing an HTC headset, the experimental process was complicated, so subjects were screened to ensure they met the experimental conditions; subjects with too much interest in the panoramic video topic or an eye movement data sampling rate lower than 80% were excluded. Finally, the number of valid subjects was 90, with 23 men and 67 women. The experiment randomly divided the 90 subjects into six groups with an average age of 21.49 years, and the subjects were paid a certain amount of money after completing the experimental process.

### 3.2 Study design

**Figure 1:**
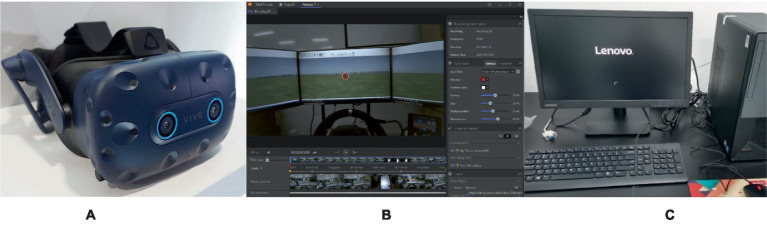
Panoramic video rendering device. **(A)** HTC VIVE PRO EYE, **(B)** tobiiProLab, **(C)** Lenovo Y720.

The study investigated the differences in participants’ attention, emotions, and experiences when viewing different panoramic videos with the same device. Subjects were divided into six groups, each of which used the same panoramic video resource, with only the caption and narration design differing across groups and the remaining picture content remaining identical. Each experimenter was randomly assigned to one of the six experimental groups. HTC VIVE PRO EYE, with clear positioning and a stable connection, was used to provide users with a good experience; a Lenovo Y720 desktop computer with a stable network was used to facilitate questionnaire data collection; and tobiiProLab was used to provide a visual user interface to effectively guide and support the researchers through all phases of the eye-tracking study, from test design to recording to analysis. Figure 1 shows the key experimental equipment used in this study.

### 3.3 Experimental materials and apparatus

#### 3.3.1 Video hardware

The experiment used the HTC VIVE PRO EYE embedded eye tracking module to enhance the VR technology experience, and a 3D stereo sound field was used. The screen was a 2 × 3.5-inch AMOLED with a 1,440 × 1,600 monocular resolution and 3 K (2,880 × 1,600) binocular resolution, a refresh rate of 90 Hz, and a field of view of 110 degrees. A Hi-Res Audio certified head unit was used for audio output. Adjustable lens distance (for users wearing glasses), adjustable pupil distance, adjustable headphones, and adjustable headband were used to ensure ergonomic design.

The stimulus material was shown with the following computer configuration: Lenovo (Lenovo) Y720 desktop computer host (CPU model: i7-7700; memory: 8G; hard disk: 1 T + 128G SSD), a Nvidia GeForce GTX 1070 8G DDR5 Solo graphics card, and a Win10operating system. The experimental stimulus presentation software fortobiiProLab was used with the built-in five-point calibration method.

#### 3.3.2 Panoramic video production

**Figure 2:**
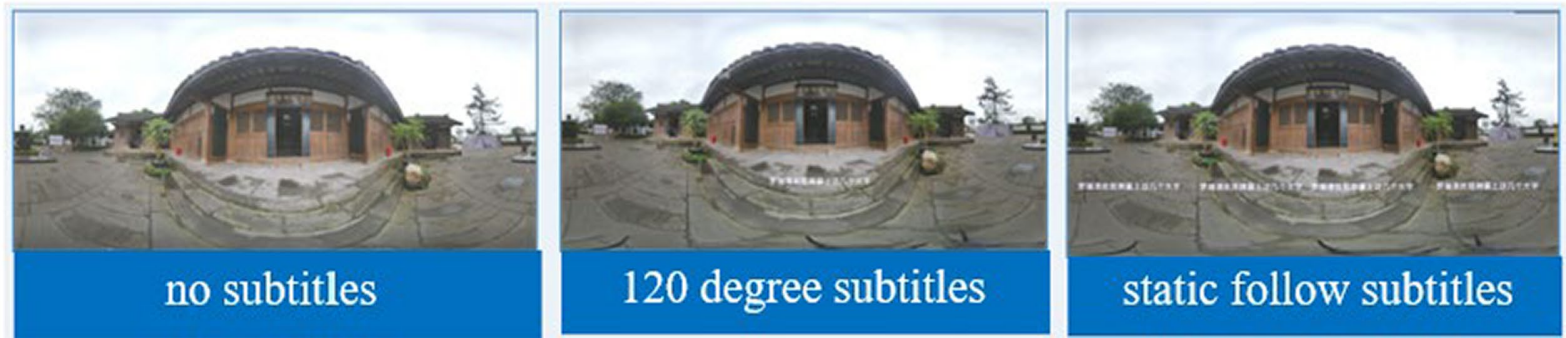
Experimental material presentation format.

The video content was recorded in panoramic shots of an exhibition room presenting a life story, a screening room with film and television materials, an exhibition room with calligraphy and painting, and a sculpture by Luo Ruiqing. The subtitles were edited and completed by Premiere, and real-voice dubbing was based on the subtitles. The AI dubbing was intelligently generated by AI in the cattle film network. The speed, intonation, and sentence length of the dubbing were intelligently controlled by the system, imported into Premiere for synthesis and production, and finally exported to an MP4 file in H.264 format for storage; the files had a duration of 3 min and 55 s. The panoramic video material is shown in Figure 2.

#### 3.3.3 Measurement methods and instruments

##### 3.3.3.1 Learning to pay attention

In this study, the eye-movement indicators of the gaze category were total fixation duration, number of fixations, and first fixation duration, and the eye-hopping indicator was the number of saccades, reflecting the learners’ attention and affective changes to the learning materials.

The total fixation duration was the sum of the durations of all the learners’ gaze points in the picture, indicating the learners’ attention to the target. The longer the total gaze time is, the higher the learners’ interest in the target content and the higher the corresponding information processing complexity (Goller et al., 2019).

The number of fixations was the sum of all the learners’ gaze points in the picture, reflecting the difficulty level of the target knowledge content.

The first fixation duration is the duration of the learner’s first gaze at the target content in the picture and is usually used to indicate the initial degree of processing of the target content (Kirkby et al., 2022).

The number of saccades was recorded, as most eye jumps are expressed as a jump from a known area to an unknown area. The higher the number of eye jumps is, the greater the degree of interference with the target content or the absence of the target content (Liechty and Madhavan, 2011).

##### 3.3.3.2 Learning emotions

Thus, emotions and learning attention are correlated, with both related to the learning experience of whether the external environment meets one’s needs. Some studies have found that emotions interfere with learning by producing irrelevant attention to the target learning content, representing an important effect of emotions on learning attention (Gong et al., 2017). Therefore, the present study used an emotion scale to measure learners’ emotions to provide a complementary account of learning attention. The emotion scale is shown in Table 2.

**Table 2:** Pre- and postmeasures of learning emotions.

Serial number	Characterization	Options				
		Hardly	Relatively little	Moderately	More often	Extremely much
1	Interested					
2	Excited					
3	Enthusiastic					
4	Inspired					
5	Determined					
6	Active					

##### 3.3.3.3 Sense of experience

In this study, to give learners a deeper sense of experience, the question items used in the sense of experience scale were modified according to the purpose of this study by referring to the research of Jennett C, Cox A L, etc. (Jennett et al., 2008).

### 3.4 Experimental steps

#### 3.4.1 Pretest

The subjects were invited to the laboratory. First, the purpose and requirements of the experiment were explained so that the subjects’ mood was relatively calm. They then completed the basic information questionnaire to report their current emotions. Subjects reported their basic information and subject interests by responding to questions such as “How old are you?,” “How interested are you in panoramic videos?,” “How interested are you in panoramic videos,” and “Are you going to study in Luo Ruiqing Memorial Hall?.” The emotional pretest included emotions: interested, excited, enthusiastic, inspired, strong-willed, and energetic. The scale used a five-point Likert scale. The laboratory environment was clean and tidy, with no overexaggerated elements to avoid ups and downs in the subjects’ mood.

#### 3.4.2 Eye movement test

**Figure 3:**
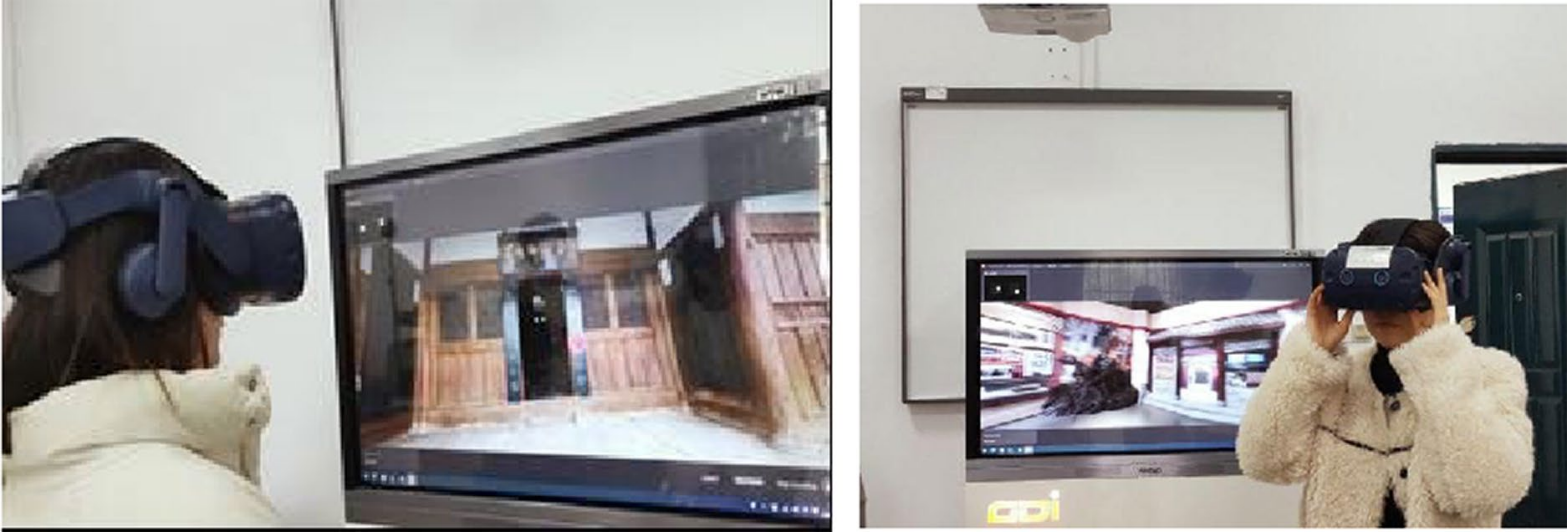
Field diagram of the panoramic video experimental process.

Before the start of the experiment, it was necessary to confirm that the subjects’ vision was normal after correction, as they were allowed to wear glasses to participate in the experiment. For the subject to adjust the HTC helmet and find the most comfortable state, the experimental stimulus presentation software Fortobii ProLab was used. Using the built-in five-point calibration method, each subject needed to be successfully calibrated before the official experimental material could be viewed. The actual panoramic video experiment is shown in Figure 3.

#### 3.4.3 Posttest

At the end of the experiment, subjects removed the helmet and expressed their experiences in a first impression interview. They then completed the posttest emotion scale.

## 4 Results

The entire experimental data collection was performed in the Tobiiprolab software, the data export was realized with the software after the experiment was finished, and the eye-movement index selection was performed with the analyze and metrics functions of the software. In this experiment, fixation samples with 80% or more valid data were selected for analysis, and the eye-movement index, which is the focus of this study, was selected and exported. The experimental group, subject number, total fixation duration, total fixation times, first fixation duration, and saccade counts were used to express learning attention.

### 4.1 Descriptive statistics

The overall eye-movement index derived after the experiments was used for two interpretation methods (AI interpretation, live interpretation) * three different caption presentation modes (no caption, 120-degree caption, static follow caption) for the six experimental groups, total fixation duration (seconds), total fixation times (times), first fixation duration (seconds), and total saccade counts (times), as shown in Table 3.

**Table 3:** Statistics of different subtitle presentation descriptions under different narration methods.

Eye movement indicator (sec/s)	Interpretation method	Presentation mode	Average	Standard deviation	*N*
Total fixation duration	AI interpretation	No subtitles	125.34	28.74	15
		120-degree caption	119.02	14.84	15
		Static follow caption	111.46	15.61	15
		Total	118.61	21.06	45
	Live interpretation	No subtitles	119.8	15.42	15
		120-degree caption	117.74	16.54	15
		Static follow caption	126.38	15.35	15
		Total	121.31	15.86	45
	Total	No subtitles	122.57	22.83	30
		120-degree caption	118.38	15.46	30
		Static follow caption	118.92	17	30
		Total	119.96	18.58	90
Number of whole fixations	AI interpretation	No subtitles	670.8	80.14	15
		120-degree caption	582.33	141.19	15
		Static follow caption	662.93	101.21	15
		Total	638.69	115.24	45
	Live interpretation	No subtitles	604.67	96.32	15
		120-degree caption	657.07	100.63	15
		Static follow caption	669.47	95.38	15
		Total	643.73	99.37	45
	Total	No subtitles	637.73	93.33	30
		120-degree caption	619.7	126.32	30
		Static follow caption	666.2	96.68	30
		Total	641.21	107.02	90
First fixation duration	AI interpretation	No subtitles	0.26	0.11	15
		120-degree caption	0.22	0.08	15
		Static follow caption	0.22	0.09	15
		Total	0.23	0.09	45
	Live interpretation	No subtitles	0.2	0.11	15
		120-degree caption	0.25	0.16	15
		Static follow caption	0.2	0.13	15
		Total	0.21	0.14	45
	Total	No subtitles	0.23	0.12	30
		120-degree caption	0.23	0.13	30
		Static follow caption	0.21	0.11	30
		Total	0.22	0.12	90
Number of saccades	AI interpretation	No subtitles	322.33	105.72	15
		120-degree caption	282.73	86.79	15
		Static follow caption	305.27	98.96	15
		Total	303.44	96.63	45
	Live interpretation	No subtitles	295.33	107.63	15
		120-degree caption	275.27	107.54	15
		Static follow caption	332.67	105.76	15
		Total	301.09	107.25	45
	Total	No subtitles	308.83	105.72	30
		120-degree caption	279	96.09	30
		Static follow caption	318.97	101.6	30
		Total	302.27	101.51	90

**Figure 4:**
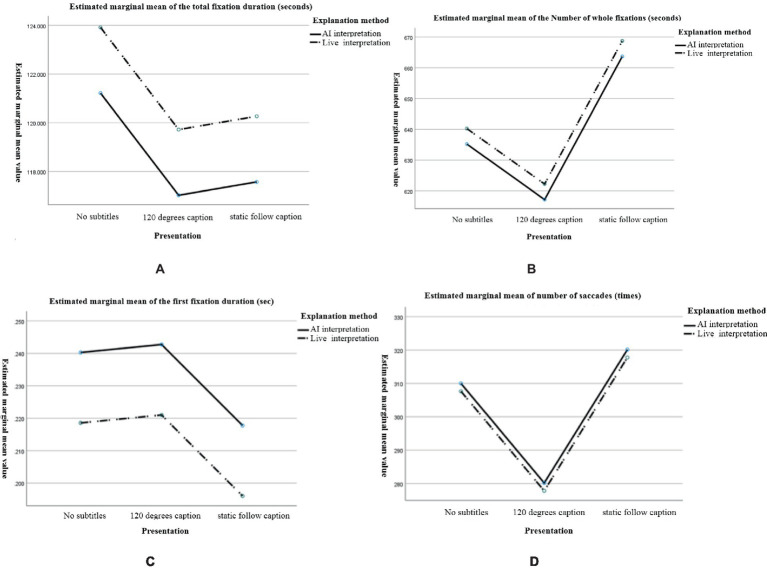
Estimated marginal mean size of eye movement metrics for different narration and subtitle presentation methods. **(A)** The total fixation duration, **(B)** total fixation times, **(C)** first fixation duration, and **(D)** total saccade counts.

In the above table, the descriptive statistics of the eye-movement index between the two interpretation methods and the three caption presentation modes are illustrated with their mean and standard deviation, and a visual contour plot is given, as shown in Figure 4.

In this experiment, the total fixation duration reflects the selective attention of the learners during the first stage of the learning process, understanding the main content. The greater the total attention time is, the higher the attention level; the less the total attention time is, the lower the attention level. The total attention time of the narration method was ranked as follows: live interpretation > AI interpretation. The total attention time of subtitle presentation was ranked as no subtitle >120-degree subtitle > static following subtitle.

In this experiment, the number of whole fixations reflects the learners’ recognition of multimedia information. If the recognition of information in the area of interest is low, then it needs to be extracted several times, and higher gaze counts indicated that the learners had more difficulty extracting the information from the experimental material; lower gaze counts indicated that it was easier for learners to extract the information from the experimental material. The total gaze number for the caption presentation was ranked as follows: static following caption >120-degree caption > no caption.

In this experiment, the first fixation duration reflects the duration of the learners’ first gaze at an object or area, and it is usually used to indicate the initial recognition of the target stimulus. Longer first gaze durations indicate higher attention levels; lower first gaze durations indicate lower attention levels. There was no significant difference in the first gaze time oculomotor data in this experiment.

In this experiment, the number of saccades reflects the guidance the multimedia learning content provided for the attention distribution of learners. More eye jumps indicated greater interference with the experimental material and greater attention levels. The number of eye jumps was ranked as follows: static following caption > no caption >120-degree caption.

### 4.2 Significance analysis

**Figure 5:**
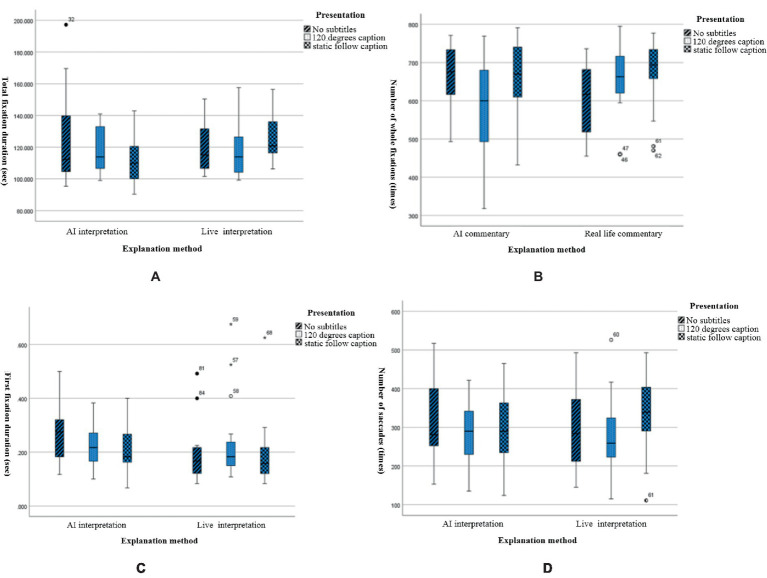
Box plots of eye-tracking indicators for different narration and subtitle presentations. **(A)** The total fixation duration, **(B)** total fixation times, **(C)** first fixation duration, and **(D)** total saccade counts.

A chi-square test and between-subjects effect test were performed on the eye-movement indicators, and box plots were used to analyze each indicator to determine whether there was a significant difference between the indicators in this study. The total gaze time data are shown in Tables 4, 5, and the box plots are shown in Figure 5.

**Table 4:** Significance statistics of eye-movement indexes.

Eye-movement indicator (sec/s)	Explanation style	Significance	Show results description
Total gaze time	explanation style	0.489 > 0.05	–
	subtitle presentation	0.633 > 0.05	–
Total number of gazes	explanation style	0.236 > 0.05	–
	subtitle presentation	0.022 < 0.05	static follow subtitles>no subtitles>120 degree subtitles
First gaze time	Explanation style	0.676 > 0.05	–
	subtitle presentation	0.014 < 0.05	No subtitles>120 degree subtitles>static follow subtitles
Number of eye jumps	Explanation style	0.933 > 0.05	-
	Subtitle presentation	0.006 < 0.05	Static follow subtitles>no subtitles>120 degree subtitles

**Table 5:** Emotional and experiential sense of significance statistics.

Type	Indicators	Significance	Show results description
Type of commentary	Emotions	0.099 > 0.05	–
	Sense of experience	0.042 < 0.05	Live interpretation > AI interpretation
Types of subtitles	Emotions	0.409 > 0.05	–
	Sense of experience	0.722 > 0.05	–

The results showed that there was no significant difference between live interpretation and AI interpretation. For the caption presentation method, the AI interpretation method had the order of no caption > static following caption >120-degree caption, and the live interpretation method had the order of static following caption > no caption >120-degree caption.

Combining the analysis of each eye-movement index in the panoramic video, the difference was not significant for learners in the interpretation method but was significant for learners in the caption presentation mode. Due to the length of the panoramic video and the large amount of eye-movement index data collected, the judgment of a small number of scenes was not analyzed in detail, so it is necessary to divide the panoramic video into an interest area and conduct an in-depth study on the representative scenes that are to facilitate determining the key learning content, from which we can obtain additional experimental data to support the research objectives.

### 4.3 Analysis of eye-movement data in the area of interest

**Figure 6:**
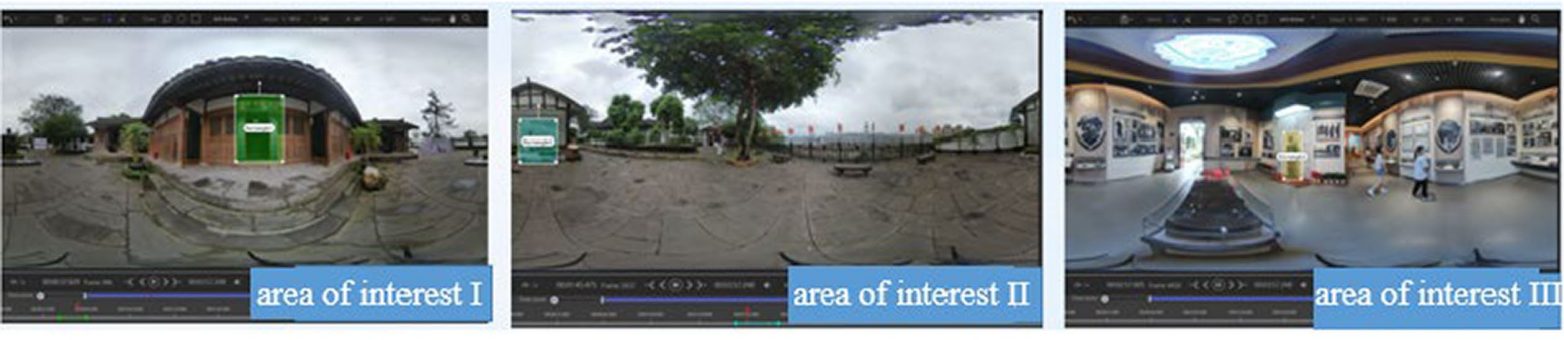
Schematic diagram of interest area division.

There were three interest areas. Area of interest I was close to the learner and introduced at the beginning of the panoramic video, with obvious markings; it was not surrounded by distractions. Area of interest II was presented far from the learner and in the “middle” of the experimental material timeline, with obvious markings and an open field of view. Area of interest III was farther away from the learner and in the “middle” of the experimental material timeline, and it was clearly marked and had a wider field of view. The areas of interest were divided as shown in Figure 6. The significance analysis of the eye-movement indexes in the three areas of interest is shown in Table 6.

**Table 6:** Significance analysis of eye-movement indexes within the area of interest.

Area of interest	Eye-Movement Indicator (sec/s)	Significance	Show results description
Area of interest I	Total fixation duration	0.01 < 0.05	–
	Number of whole fixations	0.022 < 0.05	Static follow subtitles>no subtitles>120 degree subtitles
	First fixation duration	0.014 < 0.05	No subtitles>120 degree subtitles>static follow subtitles
	Number of saccades	0.006 < 0.05	Static follow subtitles>no subtitles>120 degree subtitles
Area of interest II	Total fixation duration	0.01 < 0.05	Static follow subtitles>120 degree subtitles>no subtitles
	Number of whole fixations	0.042 < 0.05	Static follow subtitles>120 degree subtitles>no subtitles
	First fixation duration	0.242 > 0.05	–
	Number of saccades	0.026 < 0.05	Static follow subtitles>120 degree subtitles>no subtitles
Area of interest III	Total fixation duration	0.093 > 0.05	–
	Number of whole fixations	0.264 > 0.05	–
	First fixation duration	0.851 > 0.05	–
	Number of saccades	0.044 < 0.05	No subtitles>static follow subtitles>120-degree subtitles

The eye-movement data of interest areas I, II and III showed no significant difference in the narration mode, and the subtitle presentation mode varied with the scene and location of the content; that is, the subtitle presentation mode was related to the environment in which the interest area was located, the surrounding distractions and the scene of the panoramic screen, so it was necessary to consider making corresponding adjustments according to the content of different screens.

**Figure 7:**
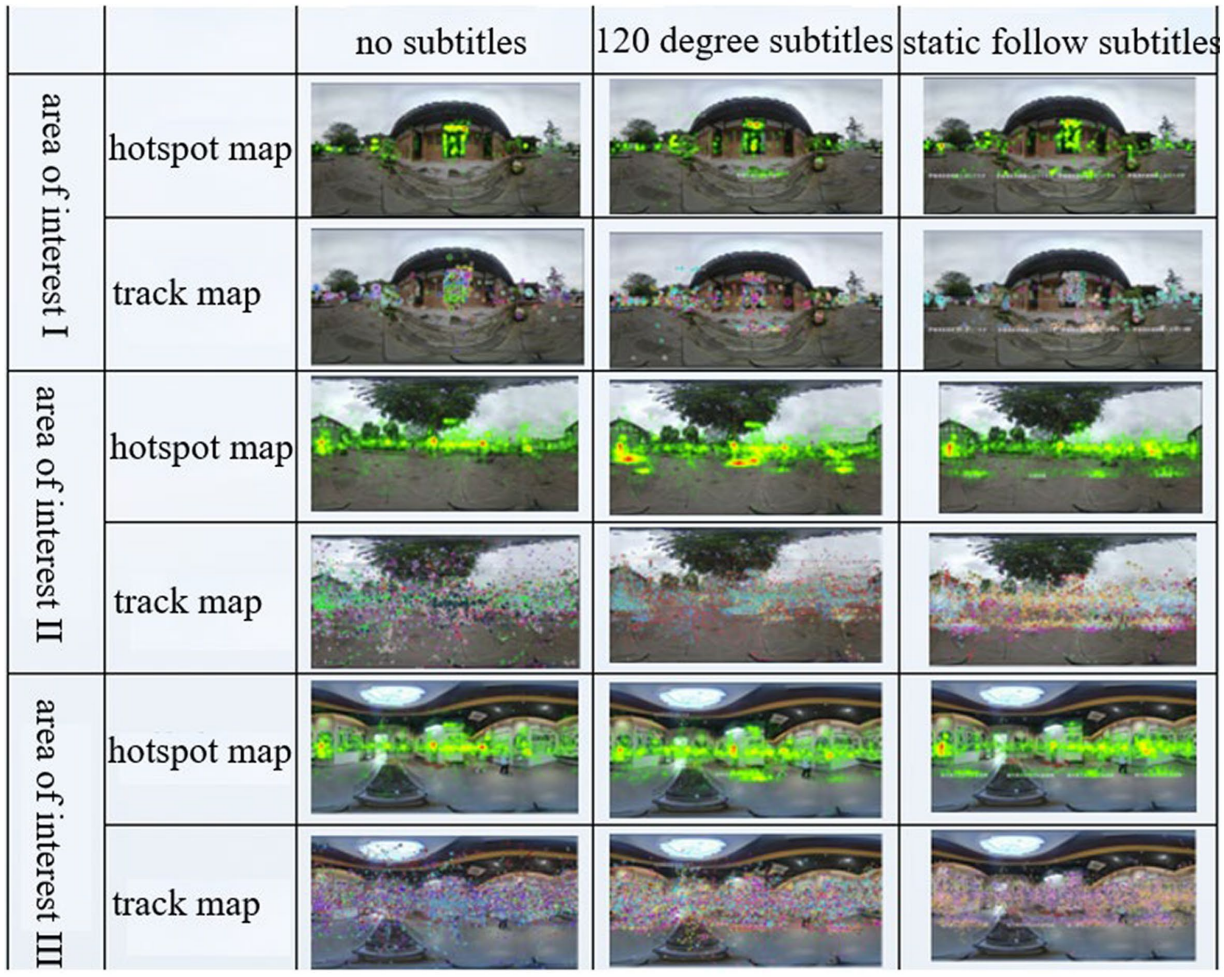
Trajectory map and hotspot map of the area of interest.

As shown in Figure 7, the combined eye-movement data of the three interest areas and the gaze hotspot map and trajectory map indicate that the way the interest area is narrated in a short period did not affect the attention to the interest area, and the caption presentation method shows that presentations without captions resulted in the most gaze points and the longest gaze times, but the gaze trajectory map was the most scattered, with more distracted attention. The 120-degree caption produced the least gaze times and the least number of gazes, probably because the caption presentation in one direction cannot attract learners’ attention to the interest area. The static following caption resulted in a concentrated distribution of gaze points, showing good attraction to learners.

### 4.4 Analysis of learners’ emotions and sense of experience

Because the scale developed in this study for the panoramic video resources improved on an existing scale, a validation factor analysis was conducted for the collected data, the KMO values and Bartlett’s sphericity test were obtained to meet the requirements of factor analysis, and a significance analysis was conducted for learning emotion and sense of experience, as shown in Table 7.

**Table 7:** Significance analysis of emotion and sense of experience in the interest zone.

Indicators	Testing	Average	Standard deviation
Emotion measurement	Pretest	3.352	0.740
	Posttest	3.829	0.390
Emotions	AI interpretation	3.902	0.089
	Live interpretation	4.111	0.089
Sense of experience	AI interpretation	3.906	0.083
	Live interpretation	4.149	0.083
Emotions	No subtitles	4.100	0.109
	120-degree caption	4.024	0.109
	Static follow caption	3.895	0.109
Sense of experience	No subtitles	3.969	0.102
	120-degree caption	4.085	0.102
	Static follow caption	4.027	0.102

In this experiment, through the analysis of the emotion scale completed by learners after experiencing the subject material, H1b was verified. When learners viewed panoramic videos, the live interpretation method resulted in better learning emotion than the AI interpretation method, and H2b was confirmed. When learners viewed panoramic videos, the caption presentation method impacted their learning emotions in the following order: no caption >120-degree caption > static follow caption. The analysis of the pretest and posttest emotion data showed that the posttest emotion was significantly higher than the pretest emotion in all experimental groups. Among the narration methods, live interpretation resulted in significantly higher emotion than the AI interpretation method, which proved that the learners’ emotions were significantly improved by this method. There was no significant difference in subtitle presentation.

In this experiment, we analyzed the experience perception scales completed by learners after experiencing the subject materials and verified Hlc. Learners reported better experiences with the panoramic video with live interpretation than with AI interpretation, confirming H2c. The order of the learners’ experiences of the panoramic video was no caption >120-degree caption > static follow caption.

## 5 Experimental results are discussed, and strategies are proposed

The main purpose of this study was to explore the effects of interpretation method and caption presentation mode on learning attention, learning mood and sense of experience when using panoramic video for learning. Through our experiments we get the following conclusion that live interpretation and caption presentation are more helpful in enhancing learners’ performance in panoramic videos. In addition, we further analyzed and proposed strategies for designing educational panoramic video screens based on our findings.

### 5.1 Discussion

#### 5.1.1 The effect of the interpretation method on learning attention, learning mood and sense of experience

To answer the first question, we analyzed whether learners’ attention (H1a), learning emotions (H1b), and senses of experience (H1c) in response to panoramic videos were affected by the mode of interpretation. Based on the results of our study, we found that participants who watched panoramic videos with interpretations by a real person outperformed learners who watched panoramic videos with interpretations by an AI in terms of learning attention, learning emotion, and a sense of experience. Consistent with our hypothesis, the use of live human interpretation in panoramic videos had a positive impact. This finding could be interpreted to indicate that humans learn better from human voices than from computer voices (the Sound principle: Mayer, 2021). Due to the lack of emotion in AI dubbing, learners’ learning emotions and senses of experience may be reduced, thus affecting their attention (Yingmin, 2023).

#### 5.1.2 The effect of caption presentation mode on learning attention, learning emotions and experiences

To answer the second question, we analyzed whether learners’ attention (H2a), learning emotions (H2b), and senses of experience (H2c) were affected by the way subtitles were presented in the panoramic video. Based on the analysis of eye-movement data and interest data, we found that overall, the no caption group showed the best results in terms of learning attention, learning emotion, and sense of experience, followed by the 120-degree caption group, and finally, the static follow caption group. The signal principle in multimedia instructional design indicates that two-dimensional video with text presentation has a better learning effect on learners than nontext presentation. In this study, it is concluded that a presentation without subtitles is better than a presentation with subtitles for three-dimensional videos (Mayer, 2021). This may be because the information presented by panoramic videos may lead to information overload, increase the cognitive load in the learning process, and affect learners’ processing and integration of learning content. This finding can be explained by the cognitive load theory (CLT) proposed by John Sweller in 1988, which suggests that learning performance is largely limited by the learner’s working memory, which can process only a limited number of information elements in a given amount of time, and that presenting information in multiple modes is disruptive to the learner when a single form is sufficient to convey the information in a concise manner. Such presentations interfere with learning, increase learners’ external cognitive loads, and reduce the quality of learning (Sweller, 1988). Furthermore, many other studies have found that videos with subtitles increase the cognitive loads of learners, distract their attention, and reduce the learning effect (Cheah and Leong, 2019; Tarchi et al., 2021).

According to Adesope and Nesbit, whether a redundancy effect occurs is moderated by factors such as the nature of the learning material, the way it is presented, and the learner’s prior experience (Adesope and Nesbit, 2012). John Sweller’s study further suggested that if cognitive information is presented rationally with the help of multiple modalities (i.e., visual and auditory), the cognitive load is reduced, and no redundancy effect occurs (Sweller, 1988). These findings indicate the existence of boundary conditions for the effectiveness of subtitling in instructional videos, thus inspiring educational researchers to streamline the form in which learning content combinations are used and to explore the matching laws of various types of media symbols. Therefore, this study further takes video points, refines the experimental groups of three subtitle presentation modes for educational panoramic videos, and constructs three interest zones with different scene environments. Through the analysis of the interest area data, the three presentation modes of no caption, 120-degree caption, and static follow caption have their own advantages for various scene types. Therefore, a design strategy for educational panoramic videos is proposed based on the results of the analysis of interest area data, with the aim of optimizing the resources and providing learners with a better learning environment.

### 5.2 Educational panoramic video screen design strategy proposed

**Figure 8:**
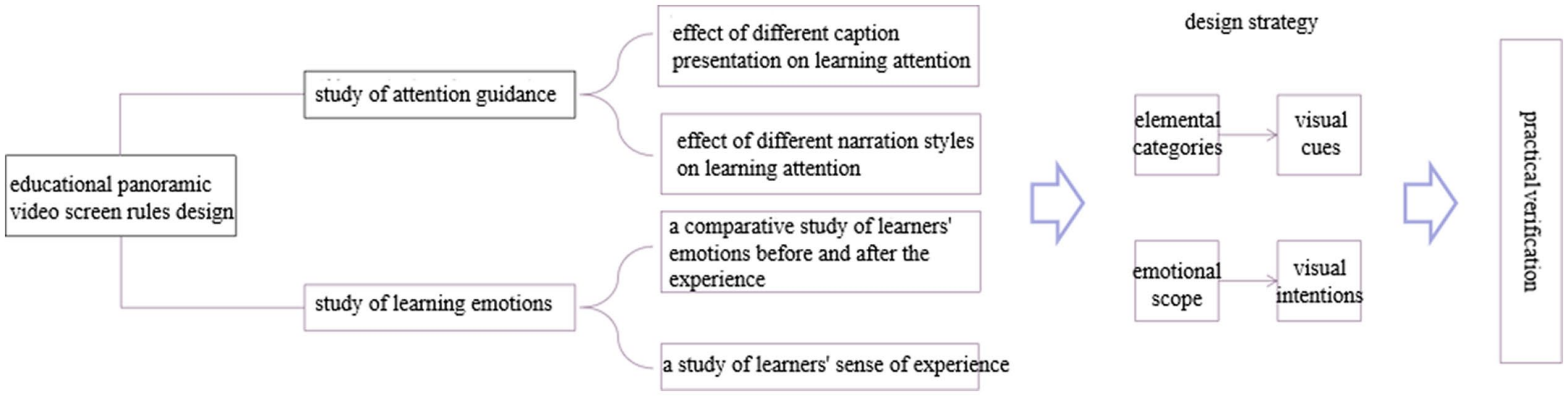
Logical relationship between experimental study and design strategy.

The development of educational panoramic videos oriented to guide attention aims to standardize the elements of educational panoramic video development and provide design ideas for educational panoramic video developers in educational technology and other fields, focusing on the use of multimedia picture language for standardized representations to generate deep cognitive activities in learners and provide guidance for teaching practice. This study uses experimental research to hypothesize and verify the two elements of captioning and narration in educational panoramic videos, and the experimental results are discussed. To this point, corresponding design strategies for optimally designing educational panoramic video screens should be proposed to increase learners’ attention. According to the conclusion of the experimental study, the design of educational panoramic video screens should be planned based on the overall design of the screen for the elemental and emotional aspects of the design strategy. The logical relationship between the experimental study and the design strategy is shown in Figure 8.

This study quantifies the caption and narration elements in the virtual words presented on a panoramic video screen through an experimental study using eye-tracking technology. The eye-tracking data show that how learners perceive the narration within panoramic videos has no significant effect on their attention to learning, which can be explained by neuroscience. The panoramic video experience begins in the visual centers of the brain, with the initial input of stimulus information coming from the eyes rather than the ears (Nevills and Wolfe, 2009). When sufficient information is transmitted to the brain through the eyes, there is an inhibitory system in the brain that allows learners to select what they think is important to learn, unlike the thalamus, which unconsciously filters out unnecessary information. Thus, learners selectively filter information processing in the auditory channel when the visual stimuli they first perceive are sufficiently jarring. The results of this study are advantageous in terms of the cost of panoramic video production. When panoramic videos are developed, there is no difference in the impact of directing the learners’ attention between live and AI narration, and to control cost, lower-cost AI interpretation can be used for production to obtain the maximum benefit. To control the learner’s experience, live interpretation can be used to make the overall picture more attractive and enjoyable for the learner.

The subtitle presentation method needs to be set according to the scene and position of the specific content. The characteristics of the three subtitle presentation methods and the eye-movement experimental data show that static follow captioning allows learners to focus their attention on the picture and can be used to illustrate the scene; that is, it can guide learners’ attention and is thus suitable for panoramic videos when the environment is relatively open. It is recommended that, for outdoor scenes with iconic blur that require guidance, 120-degree captioning enables more attention points in the key images, which is suitable for annotating the key learning contents and guiding learners to the key knowledge points more quickly. For indoor venue scenes that need to be explained, visual attention is more dispersed without captioning, enabling learners more space to view the scenes. Panoramic video opening scenes do not need to be explained. Notably, individual differences in learners and subjective picture element preferences may be important factors in determining learners’ attention, and further verification is needed.

In terms of emotion, panoramic video images should be based on learners’ experiences, and to enable learners to form correct visual impressions, the knowledge content conveyed by panoramic video images must be correct and reflect the essential state of things in a real and objective way and avoid too many late “virtual” elements that increase learners’ visual loads and thus generate discomfort.

## 6 Conclusion and future research

In this study, we used motion-tracking technology to investigate the effects of narration (AI vs. live interpretation) and captioning (no captioning, 120-degree captioning, and static follow captioning) on learners’ attention, emotions, and experiences toward the components of panoramic video development. The results show that in terms of learners’ attention, emotions and experiences, live interpretation is better than AI interpretation, no captions are better than 120-degree captions, and 120-degree captions are better than static follow captions in educational panoramic videos. For the different environments for learning content, we further found that each of these subtitle presentation methods has its own advantages in different scenarios and proposed panoramic video design strategies for specific learning scenarios.

This study extends parts of multimedia learning theory to virtual learning environments and proposes a reasonable strategy for designing educational panoramic videos. However, there are several limitations to this research. First, it was a short-term study with a limited sample size conducted in a laboratory setting, and the validity of the results needs to be tested in a real learning environment. Second, because of time and experimental scale limitations, this study mainly explored the effects of narration and subtitle presentation on learners’ attention, learning emotions and senses of experience in the components of panoramic video development and did not consider the elements of color, light, composition, location, or animation in depth. Third, the study material has some limitations. This study used a certain chapter of a course in education as the experimental content and was not extended to other disciplines, and differences across disciplines may also affect the results of the experiment. Finally, this study controlled for factors such as learner major, age, visual acuity, and prior knowledge but did not discuss the interactions among learners’ individual characteristics, subjective picture element preferences and the study’s independent variables (narration style and subtitle presentation).

Based on the results and limitations of the current study, we propose the following aspects for further research:

(1) Future research should employ larger samples and consider more realistic learning environments.
(2) To offset the limitations of time and scale, future research should investigate and study other elements in panoramic virtual learning environments, such as color, light, composition, and location.
(3) Future research should consider more types of learning materials and determine whether differences in subject content affect experimental effects.
(4) The role of learners’ individual characteristics and subjective screen element preferences (learning style, learning motivation, etc.) in relation to the narration and subtitle presentation should also be studied.

## Data availability statement

The original contributions presented in the study are included in the article/supplementary material, further inquiries can be directed to the corresponding author.

## Ethics statement

The studies involving humans were approved by Ethics Committee of China West Normal University. The studies were conducted in accordance with the local legislation and institutional requirements. The patients/participants provided their written informed consent to participate in this study.

## Author contributions

GH: Funding acquisition, Project administration, Resources, Supervision, Writing – review & editing, Conceptualization. LZ: Conceptualization, Data curation, Investigation, Methodology, Visualization, Writing – original draft, Writing – review & editing, Validation. DC: Conceptualization, Data curation, Software, Writing – review & editing. WC: Funding acquisition, Methodology, Resources, Visualization, Writing – review & editing. RL: Software, Supervision, Validation, Writing – review & editing.
